# Depletion of Endothelial or Smooth Muscle Cell-Specific Angiotensin II Type 1a Receptors Does Not Influence Aortic Aneurysms or Atherosclerosis in LDL Receptor Deficient Mice

**DOI:** 10.1371/journal.pone.0051483

**Published:** 2012-12-07

**Authors:** Debra L. Rateri, Jessica J. Moorleghen, Victoria Knight, Anju Balakrishnan, Deborah A. Howatt, Lisa A. Cassis, Alan Daugherty

**Affiliations:** 1 Saha Cardiovascular Research Center, University of Kentucky, Lexington, Kentucky, United States of America; 2 Department of Molecular and Biomedical Pharmacology, University of Kentucky, Lexington, Kentucky, United States of America; Baker IDI Heart and Diabetes Institute, Australia

## Abstract

**Background:**

Whole body genetic deletion of AT1a receptors in mice uniformly reduces hypercholesterolemia and angiotensin II-(AngII) induced atherosclerosis and abdominal aortic aneurysms (AAAs). However, the role of AT1a receptor stimulation of principal cell types resident in the arterial wall remains undefined. Therefore, the aim of this study was to determine whether deletion of AT1a receptors in either endothelial cells or smooth muscle cells influences the development of atherosclerosis and AAAs.

**Methodology/Principal Findings:**

AT1a receptor floxed mice were developed in an LDL receptor −/− background. To generate endothelial or smooth muscle cell specific deficiency, AT1a receptor floxed mice were bred with mice expressing Cre under the control of either Tie2 or SM22, respectively. Groups of males and females were fed a saturated fat-enriched diet for 3 months to determine effects on atherosclerosis. Deletion of AT1a receptors in either endothelial or smooth muscle cells had no discernible effect on the size of atherosclerotic lesions. We also determined the effect of cell-specific AT1a receptor deficiency on atherosclerosis and AAAs using male mice fed a saturated fat-enriched diet and infused with AngII (1,000 ng/kg/min). Again, deletion of AT1a receptors in either endothelial or smooth muscle cells had no discernible effects on either AngII-induced atherosclerotic lesions or AAAs.

**Conclusions:**

Although previous studies have demonstrated whole body AT1a receptor deficiency diminishes atherosclerosis and AAAs, depletion of AT1a receptors in either endothelial or smooth muscle cells did not affect either of these vascular pathologies.

## Introduction

There is substantial and consistent literature demonstrating that manipulation of the renin angiotensin system has profound effects on experimental atherosclerosis and abdominal aortic aneurysms (AAAs). All these effects of the renin angiotensin system are assumed to be mediated via the major bioactive peptide of this system, angiotensin II (AngII). For atherosclerosis, pharmacological inhibition of AngII synthesis, through inhibition of ACE or renin, reduced the size of atherosclerotic lesions in several experimental models of atherosclerosis. For example, ACE inhibitors reduced atherosclerotic lesion size in mice, hamsters, rabbits, and monkeys [1]–[4]. Renin inhibition also markedly attenuated atherosclerotic lesion size in mouse and rabbit models [5]–[7]. Additionally, AngII has been invoked as a mediator of AAAs based on many studies demonstrating that chronic infusion of AngII leads to pronounced aortic expansion in hyper- and normocholesterolemic male mice [8]–[10].

The majority of the physiological and pathological effects of AngII are via stimulation of AT1 receptors [11]. Inhibition of AT1 receptors using multiple members of the sartan family of drugs profoundly reduced atherosclerosis in mouse, rabbit, and monkey models of the disease [1], [12], [13]. In mice, chromosomal duplication has resulted in expression of two isoforms of this receptor, termed AT1a and AT1b. Although these two receptors are 94% amino acid homologous and not discriminated by sartans, they have distinct patterns of distributional and functional characteristics, with the AT1a isoform being considered as the primary regulator of most AngII effects. In agreement with pharmacological inhibition, genetically engineered deletion of AT1a receptors strikingly reduced atherosclerosis in both apoE −/− and LDL receptor −/− mice in multiple studies [14]–[19]. These major effects of AT1a receptor depletion on atherosclerosis occurred without changes of pronounced hypercholesterolemia. AT1a receptor stimulation is also a requirement for the development of AngII-induced AAAs [18], [20].

While there is consistent evidence that AT1a receptors regulate the development of atherosclerotic lesions and AAAs, these effects may be attributable to stimulation of several cell types. Bone marrow transplantation has been used as an approach to define effects of AT1a receptor activation on leukocytes. However, contrary to the consistently large reductions of atherosclerosis in mice with whole body deficiency of AT1a receptors, depletion of this receptor in bone marrow-derived cells has highly variable effects. This has included a range of influences on lesion size including no effects in LDL receptor −/− and apoE−/− mice fed saturated fat enriched diets with or without AngII infusion [5], [18], [21], decreases [17], [22], and increases [23]. The sole study in AngII-induced AAAs failed to detect any effect of AT1a receptor expression in bone marrow-derived cells on development of AAAs [18]. Hence, it is unclear which cell type is being stimulated by AngII to promote the development of these two vascular pathologies.

There are many AngII-induced processes defined in cultured endothelial and smooth muscle cells, inferring its relevance to atherogenesis. However, the role of AngII stimulation of specific cell types in vivo on the development of atherosclerotic lesions and AAAs has not been directly examined. Therefore, the novelty of this study was to use our recently developed AT1a receptor floxed mice to determine the contribution of AT1a receptor stimulation in either endothelial or smooth muscle cells. Despite validation of AT1a receptor depletion in these two cell types in vivo, the absence of this receptor type in either cell type had no discernible effect on the development of atherosclerosis or AAAs.

## Materials and Methods

### Ethics Statement

This study followed the recommendations of The Guide for the Care and Use of Laboratory Animals (National Institutes of Health). All procedures were approved by the University of Kentucky’s Institutional Animal Care and Use Committee (Protocol # 2006–0009). The mice were observed daily for any signs of distress and weighed weekly to monitor health.

### Mice

Agtr1a receptor^flox/flox^ mice were generated by inGenious Targeting Laboratory using a C57BL/6 embryonic stem cell line [24]. These mice are now available at The Jackson Laboratory (C57BL/6N-*Agtr1a^tm1Uky^*/J; stock # 016211). LDL receptor −/− (stock # 2207), SM22-Cre (stock # 4746) and Tie2-Cre (stock # 4128) mice were purchased from The Jackson Laboratory (Bar Harbor, ME). SM22-Cre mice, initially acquired in a mixed background, were bred into a C57BL/6 strain and screened using The Jackson Laboratory’s Speed Congenic Service to expedite the development of the C57BL/6 background. Mice were bred to produce the following breeding harems: male Agtr1a receptor^flox/flox^ x Tie2-Cre or SM22-Cre x LDL receptor −/− and female Agtr1a receptor^flox/flox^ x LDL receptor −/− mice.

### Atherosclerosis and Aneurysm Studies

For hypercholesterolemia-induced atherosclerosis studies, male and female mice (8–12 weeks old) were fed a diet enriched in saturated fat (Diet# TD.88137; Harlan Teklad, Indianapolis, IN) containing milk fat (21% wt/wt) and cholesterol (0.2% wt/wt) for 12 weeks.

For AngII-induced atherosclerosis and aortic aneurysm studies, male mice (8 weeks old) were fed the saturated fat enriched diet listed above for 5 weeks. After 1 week of feeding, mini osmotic pumps (Model # 2004; Durect Corp, Cupertino, CA) filled with saline or AngII (1,000 ng/kg/min; Cat # H-1705; Bachem, Torrance, CA) were implanted subcutaneously in the right flanks of mice [8], [25]. Duration of continuous infusion was 28 days.

### Genotyping

Genomic DNA was isolated from mouse tails and genotyped using PCR as described previously [24]. A representative electrophoretic gel of the amplicons is shown in Figure S1.

### Serum analyses

At termination, blood was collected by cardiac puncture and serum was separated by centrifugation. Lipoprotein fractions were resolved by size exclusion chromatography from serum of individual mice (50 µl) using a size exclusive chromatography (FPLC) [26]. Eluted fractions and total serum cholesterol were measured using an enzymatic kit (Cat # Cholesterol E 439-17501; Wako Chemicals, Richmond, VA).

### Systolic Blood Pressure Measurements

Systolic blood pressure was measured on conscious mice using a noninvasive tail-cuff system (CODA 8; Kent Scientific Corp, Torrington, CT) as described previously [27]. Systolic blood pressure was determined by measurements on at least 3 consecutive days at baseline, and during the last week of study.

### Atherosclerotic Lesion and AAA Quantification

After exsanguination, aortas were perfused with saline, dissected free, and fixed in 10% neutral buffered formalin overnight. The next day, aortas were transferred to saline and adventitia were removed. For atherosclerotic lesion measurements, aortas were cut open, pinned, and photographed, and area was measured using ImagePro Plus software [28], [29]. Measurement of aortic lumen diameter was performed using a Visualsonics Vevo 660 high frequency ultrasound machine as described previously [30]. For aneurysm quantification, external diameter of suprarenal aortas was measured using ImagePro Plus software [18], [31].

### Statistical Analyses

Appropriate analyses were conducted based on group number compared and parametric characteristics of the data using SigmaPlot version 12.0 (Systat Software Inc, San Jose, CA). Data are represented as mean ± SEM. *P*<0.05 was considered statistically significant.

## Results

### AT1a Receptor Depletion in Endothelial Cells had no Effect on Hypercholesterolemia-induced Atherosclerosis

**Figure 1:**
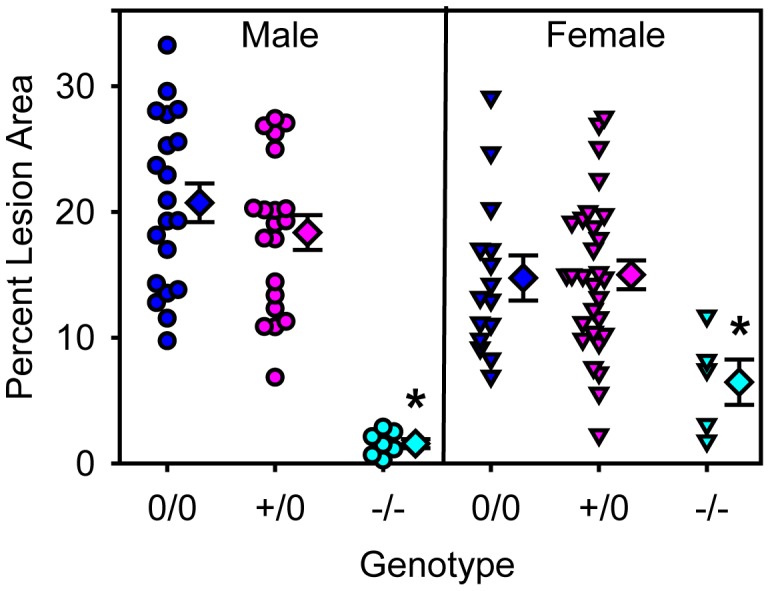
Endothelial depletion of AT1a receptors had no effects on atherosclerotic lesion area. Individual measurements are represented from littermates that were either male (circles) or female (inverted triangles). Blue symbols are non-transgenic, pink symbols are Tie2-Cre +/0, and teal symbols are whole body AT1aR deficient mice. Diamond symbols are group means and bars are standard error of means (0/0 =  non-transgenic littermate; +/0 =  Tie2-Cre hemizygous transgenic; −/− = AT1a receptor −/−). * denotes *P*<0.05 when comparing −/− to 0/0 or +/0 within either gender.

To examine the role of endothelial cell AT1a receptors in atherosclerosis, LDL receptor deficient mice were developed with floxed AT1a receptor mice that express Cre under the control of the Tie2 promoter. We have described previously that expression of Cre under control of the Tie2 promoter leads to profound reductions in AT1a receptor mRNA in endothelial cells and had consistent effects on aortic endothelial cells throughout the aorta [24]. Male and female AT1aR^flox/flox^ x LDL receptor −/− mice that were hemizygous for Cre were compared to littermates that did not express Cre. Whole body AT1a receptor deficient mice were also added as a control group. All mice were fed a diet enriched in saturated fat for 12 weeks. As expected, body weight was affected by gender as male mice were heavier than females (*P*<0.001; Table 1). Total serum cholesterol concentrations and lipoprotein distribution of cholesterol were similar in all groups (Table 1 and Figure S2). Consistent with previous publications [15], percent atherosclerotic lesion area was decreased in LDL receptor −/− mice with whole body deficiency of AT1a receptors. However, lesion areas were not significantly different between wild type and endothelial cell specific AT1a receptor deficient mice in either gender (Figure 1).

**Table 1:** Body weight and serum cholesterol concentration of LDL receptor −/− mice that were Tie2-Cre 0/0, Tie2-Cre +/0, or AT1a receptor −/−.

Genotype	Gender	n	Body weight (g)	Cholesterol (mg/dl)
Tie2-Cre 0/0	Male	20	46.7±1.4	1676±63
Tie2-Cre +/0		20	43.3±1.1	1677±95
−/−		7	36.5±2.8*	1679±118
Tie2-Cre 0/0	Female	15	34.0±1.5*	1545±71
Tie2-Cre +/0		29	34.6±1.1*	1676±59
−/−		5	27.3±1.8*	1420±204

Body weight and serum cholesterol concentration were measured at experimental termination. There were no differences in measurements between Tie2 0/0 or +/0 attained statistical significance.

* denotes *P*<0.05 compared to male within genotype (0/0 =  no Cre; +/0 =  hemizygous Cre; −/− = whole body deficiency of AT1a receptor).

### AT1a Receptor Depletion in Smooth Muscle Cells had no Effect on Hypercholesterolemia-induced Atherosclerosis

**Figure 2:**
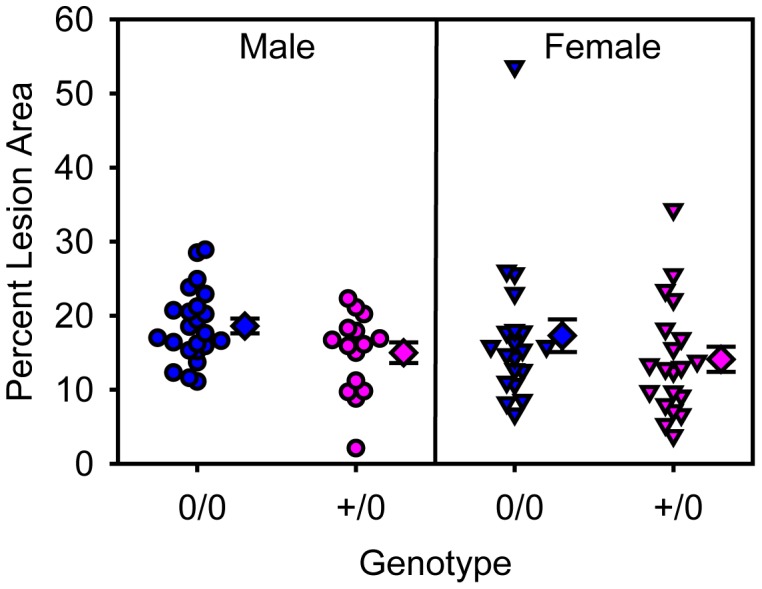
Smooth muscle cell depletion of AT1a receptors had no effects on atherosclerotic lesion area. Individual measurements are represented from littermates that were either male (circles) or female (inverted triangles). Blue symbols are non-transgenic and pink symbols are SM22-Cre +/0 mice. Diamond symbols are group means and bars are standard error of means (0/0 =  non-transgenic littermate and +/0 =  SM22-Cre hemizygous transgenic).

To examine the role of smooth muscle cell AT1a receptors in atherosclerosis, LDL receptor deficient mice were developed with floxed AT1a receptor mice that express Cre under the control of the SM22 promoter. We have described previously that the expression of Cre under the control of the SM22 promoter leads to pronounced reductions in AT1a receptor mRNA in smooth muscle cells, and has a consistent effect on smooth muscle cells throughout the aorta [24]. Male and female littermate LDL receptor −/− mice that were wild type or deficient in smooth muscle cell AT1a receptors were fed the saturated fat-enriched diet for 12 weeks. Serum total cholesterol concentrations were increased in males compared to females (male versus female: 1860±60 versus 1437±54 mg/dl; *P*<0.001), although distributions of cholesterol among lipoprotein fractions were similar between genders (Figure S3). Smooth muscle cell deficiency of AT1a receptors had no effect on serum cholesterol concentrations (Table 2). Percent atherosclerotic lesion area was equivalent between genders, and was not influenced between the genotypes of AT1a receptors in smooth muscle cells (Figure 2).

**Table 2:** Body weight and serum cholesterol concentration of LDL receptor −/− mice that were SM22-Cre 0/0 or +/0.

Cre genotype	Gender	n	Body weight (g)	Cholesterol (mg/dl)
SM22-Cre 0/0	Male	23	44.8±0.9	1943±99
SM22-Cre +/0		15	44.7±1.4	1777±94
SM22-Cre 0/0	Female	23	32.3±1.2*	1519±81*
SM22-Cre +/0		23	32.4±1.0*	1355±59*

Body weight and serum cholesterol concentration were measured at experimental termination. There were no significance differences in these measurements between SM22 0/0 or +/0 attained statistical significance.

* denotes *P*<0.05 compared to male within genotype (0/0 =  no Cre; +/0 =  hemizygous Cre).

### AT1a Receptor Depletion in Endothelial Cells had no Effect on AngII-induced AAAs and Atherosclerosis

**Figure 3:**
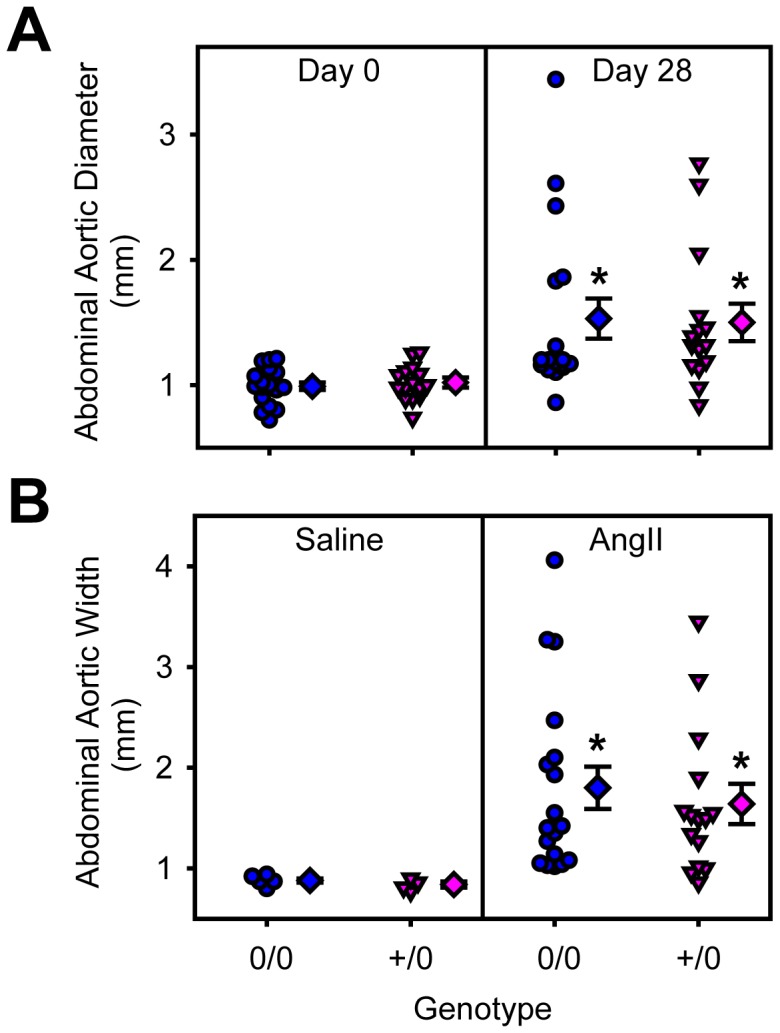
Endothelial depletion of AT1a receptors had no effects on AngII-induced abdominal aortic dilation measured *in vivo* by ultrasonography or *ex vivo*. A. Measurements of lumen diameters of suprarenal aortas by high frequent ultrasound on day 0 and day 28 of AngII infusion. B. Measurements of maximal external diameter of suprarenal aortas on *ex vivo* tissues acquired at termination. Individual measurements are represented from littermates that were either non-transgenic (circles) or Tie2-Cre +/0 (inverted triangles) infused with saline (left) or AngII (right). Diamond symbols are group means and bars are standard error of means (0/0 =  non-transgenic littermates and +/0 =  Tie2-Cre hemizygous transgenic). * denotes *P*<0.05 when comparing saline versus AngII-infused mice.

**Figure 4:**
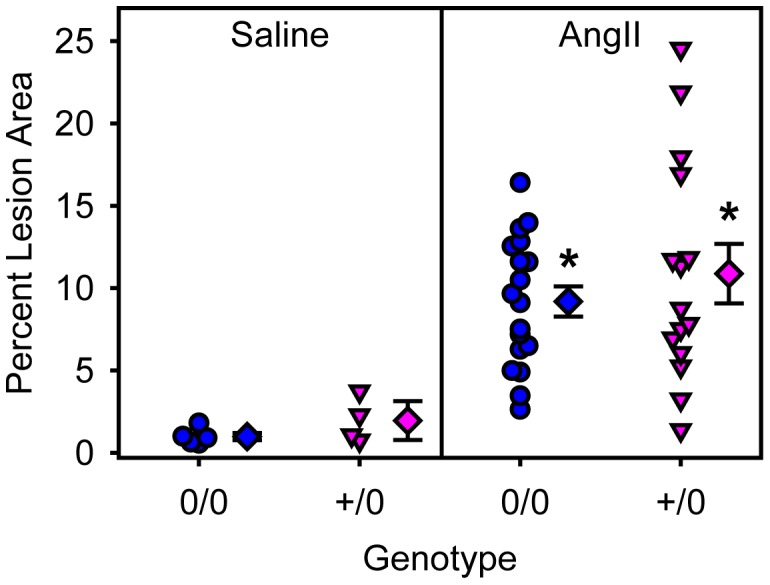
Endothelial depletion of AT1a receptors had no effects on AngII-induced atherosclerosis. Individual measurements are represented from littermates that were either non-transgenic (circles) or Tie2-Cre +/0 (inverted triangles) infused with saline (left) or AngII (right). Diamond symbols are group means and bars are standard error of means (0/0 =  non-transgenic littermate and +/0 =  Tie2-Cre hemizygous transgenic). * denotes *P*<0.05 comparing saline versus AngII infused mice.

To examine the role of endothelial cell AT1a receptors in AngII-induced AAAs and atherosclerosis, male littermate LDL receptor −/− mice, that were wild type or deficient in endothelial cell AT1a receptors, were fed a saturated fat enriched diet and infused with either saline or AngII (1,000 ng/kg/min) for 28 days. Genotype or AngII infusion had no effect on body weight and serum cholesterol concentrations (Table 3). Systolic blood pressure was not different between the saline-infused mice. AngII infusion at 1,000 ng/kg/min significantly increased systolic blood pressure in both genotypes (*P*<0.05; Table 3). Diameters of abdominal aortas were measured both *in vivo* by ultrasound and *ex vivo*. AngII infusion significantly increased lumen diameter of abdominal aortas (*P*<0.001; Figure 3A) and ex vivo aortic width (P = 0.004; Figure 3B) in both genotypes. There were no differences between genotypes (P = 0.995 and P = 0.717, respectively). AngII infusion significantly increased atherosclerosis in both genotypes when compared to saline infusion (*P*<0.001; Figure 4), whereas endothelial specific AT1a receptor deficiency did not exert any significant effect (*P* = 0.479).

**Table 3:** Body weight, serum cholesterol concentration, and systolic blood pressure of LDL receptor −/− mice with deletion of AT1a receptors in endothelial (Tie2-Cre +/0) or smooth muscle cells (SM22-Cre +/0).

Cre genotype	Infusion	n	Body weight (g)	Cholesterol (mg/dl)	SBP (mmHg)
0/0	Saline	5	30.0±0.6	1308±41	146±5
	AngII	18	30.4±0.8	1254±59	173±4*
Tie2-Cre +/0	Saline	4	28.8±1.0	1269±193	144±10
	AngII	15	29.4±1.1	1305±62	174±6*
SM22-Cre +/0	Saline	6	29.6±1.6	1269±79	136±10
	AngII	25	29.0±0.6	1561±60	152±4*

Body weight and serum cholesterol concentration were measured at experimental termination. Systolic blood pressure (SBP) was measured daily for at least 3 consecutive days during the final week of AngII infusion.

* denotes *P*<0.05 compared to saline within genotype (0/0 =  no Cre; +/0 =  hemizygous Cre).

### AT1a Receptor Depletion in Smooth Muscle Cells had no Effect on AngII-induced AAAs and Atherosclerosis

**Figure 5:**
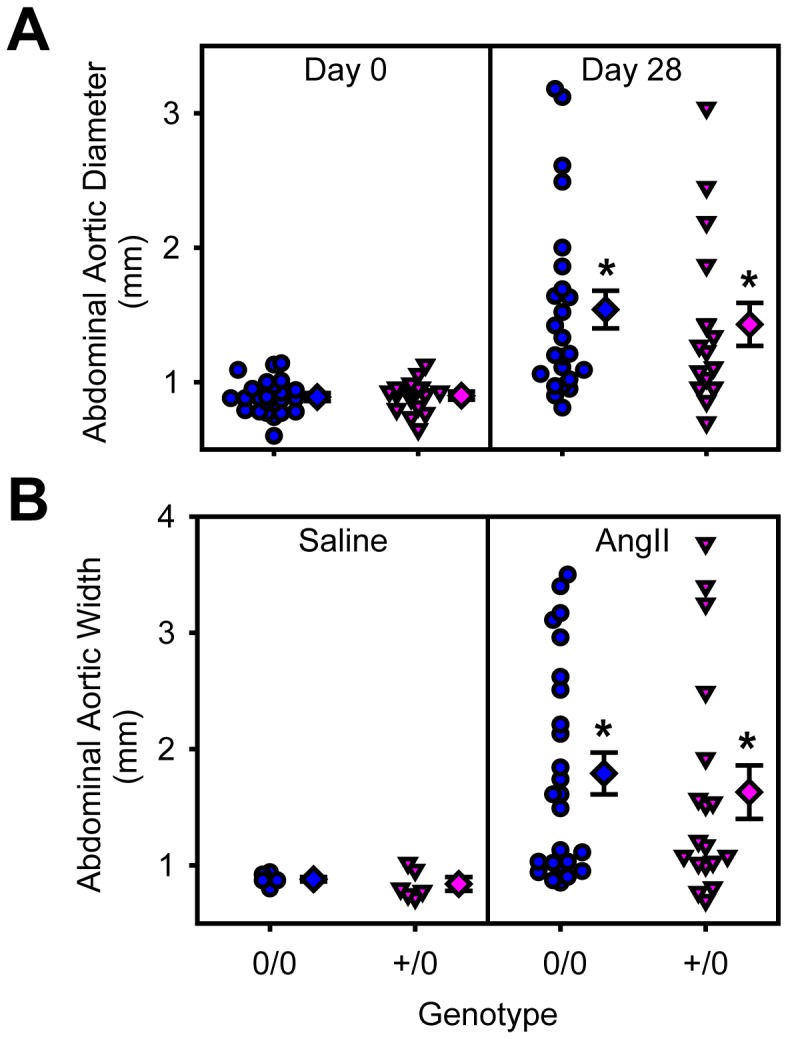
Smooth muscle cell depletion of AT1a receptors had no effects on AngII induced abdominal aortic dilation measured *in vivo* by ultrasonography or *ex vivo*. A. Individual measurements of lumen diameter of suprarenal aortas by high frequently ultrasound on day 0 and day 28 of AngII infusion. B. Individual measurements of maximal external diameter of suprarenal aortas on *ex vivo* tissue acquired a termination. Measurements are represented from littermates that were either non-transgenic (circles) or SM22-Cre +/0 (inverted triangles) infused with saline (left) or AngII (right). Diamond symbols are group mean and bars are standard error of mean (0/0 =  non-transgenic littermates and +/0 =  SM22-Cre hemizygous transgenic). * denotes *P*<0.05 comparing saline versus AngII-infused mice.

**Figure 6:**
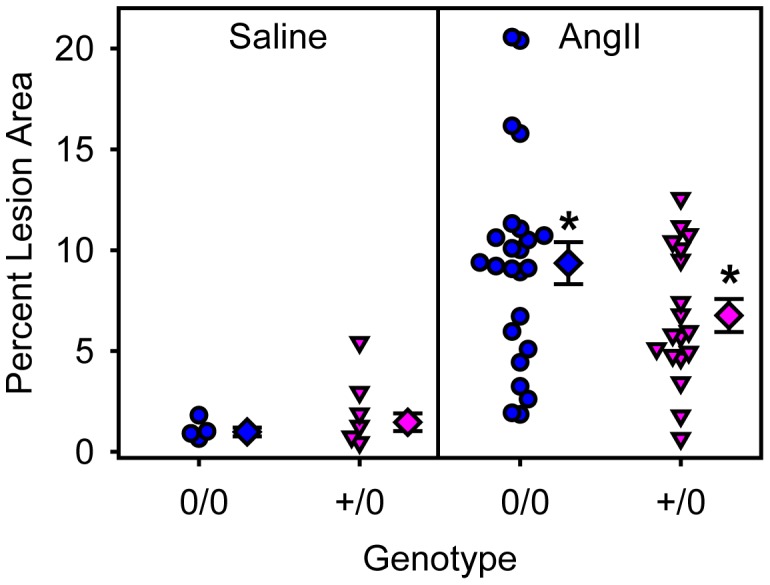
Smooth muscle cell depletion of AT1a receptors had no effects on AngII-induced atherosclerosis. Individual measurements are represented from littermates that were either non-transgenic (circles) or SM22-Cre +/0 (inverted triangles) infused with saline (left) or AngII (right). Diamond symbols are group means and bars are standard error of means (0/0 =  non-transgenic littermate and +/0 =  SM22-Cre hemizygous transgenic). * denotes *P*<0.05 comparing saline versus AngII-infused mice.

To examine the role of smooth muscle cell AT1a receptors in AngII-induced AAAs and atherosclerosis, male littermate LDL receptor −/− mice that were wild type or deficient for smooth muscle cell AT1a receptors, were fed a saturated fat-enriched diet and infused with either saline or AngII (1,000 ng/kg/min) for 28 days. Genotype or AngII infusion had no effect on body weight and serum cholesterol concentrations (Table 3). Systolic blood pressure was not different between the saline-infused mice. AngII infusion significantly elevated systolic blood pressure in both genotypes (*P*<0.05; Table 3). Abdominal aortas were scanned by ultrasound at baseline, day 0 and again at day 28 of infusion. AngII infusion significantly increased lumen diameter of abdominal aortas (*P*<0.001; Figure 5A) and ex vivo aortic width (*P* = 0.004; Figure 5B) in both genotypes. There were no differences between genotypes (*P* = 0.665 and *P* = 0.725, respectively). AngII infusion significantly increased atherosclerosis in both genotypes when compared to saline infusion (*P*<0.001; Figure 6), but similar to endothelial cell-specific deficiency, smooth muscle cell-specific AT1a receptor genotypes did not exert any significant effect (*P* = 0.459).

## Discussion

Inhibition of AT1 receptor stimulation through pharmacological antagonism or genetic manipulations has been a highly consistent mode of decreasing experimental atherosclerosis in many animal models [32]. These pronounced reductions in atherosclerotic lesion size have occurred in the absence of any discernible effect on hypercholesterolemia, which is considered a pivotal determinant of atherosclerosis [14], [15]. In agreement with this literature, the present study confirmed that effects of whole body depletion of AT1a receptors profoundly inhibited atherosclerotic formation in both genders. Inhibition of AT1 receptors also ablates the development of AngII-induced AAAs. Since endothelial and smooth muscle cells are the major resident cell types in the arterial regions of atherosclerosis and AAAs, we proposed that depletion of AT1a receptors in either of these two cell types would reduce the development of these vascular pathologies. Using AT1a receptor floxed mice with previously validated approaches to deleting AT1a receptor expression in endothelial or smooth muscle cells, we were surprised that deletion of this receptor in either of the two resident cell types had no significant effects on atherosclerosis and AAAs.

Although the effects of AT1a receptor expression on cell types resident in the arterial wall have not been determined previously, several studies have focussed on the role of AT1a receptor expression in leukocytes in the development of atherosclerosis in hypercholesterolemic mice, including some studies with AngI infusion. The abundance of leukocytes in experimental atherosclerotic lesions provides a basis for a focus on this group of cell types. The studies have used the technique of repopulating irradiated mice with bone marrow-derived cells that are either wild type or deficient in AT1a receptors. These studies have generated highly variable results that cover the spectrum of inhibiting, having no effect, or promoting atherosclerosis [5], [17], [18], [21]–[23]. We have been unable to distinguish the basis for these highly variable findings. Only one study reported the effects of leukocyte AT1a receptors on AAAs, which failed to demonstrate an effect of AT1a receptors in bone marrow-derived cells on the formation of AAAs [18]. It has been documented that the process of bone marrow transplantation may influence atherosclerosis and AAA formation in mice [18], [33]. Therefore, future studies on the role of AT1a receptor expression in leukocytes may be benefited from the application of Cre-lox approaches as performed on the cell types that were the focus of the present study.

Chronic subcutaneous infusion of AngII into hyper- or normocholesterolemic mice has been used in numerous studies to generate AAAs [8], [25], [34]. The pivotal role of AT1a receptors has been demonstrated by the ablation of AAAs in whole body AT1a receptor deficient mice [18]. Although leukocyte infiltration is a prominent feature of AngII-induced AAAs [35], deficiency of AT1a receptors in this cell type had no effect on the extent of aortic dilation [18]. Endothelial and smooth muscle cells are other potential cell types stimulated by AngII in the evolution of aneurysmal diseases. However, similar to atherosclerosis, deletion of AT1a receptors in either endothelial or smooth muscle cells had no effect on the development of AAAs.

One potential explanation for the lack of effects of AT1a receptor depletion in either endothelial or smooth muscle cells on atherosclerosis and AAAs would be limited excision of the exon 3 of the AT1a receptor gene in Cre expressing floxed mice. Using lineage tracking, we have demonstrated previously the uniformity of the AT1a receptor gene deletion in smooth muscle and endothelial cells using SM22 and Tie2 promoters, respectively, to drive Cre expression [24]. Furthermore, we have demonstrated previously that Cre driven by these promoters leads to ablation of AT1a receptor mRNA abundance in these respective cell types [24]. It would be desirable to have performed immunostaining of AT1a receptors to demonstrate deletion in these two cell types. Unfortunately, following validation with control experiments, we have been unable to demonstrate specific staining for AT1a receptors using a number of antibodies, [24], [36], [37]. The difficulty of detecting AT1a receptors in mice is compounded by the close structural similarities of AT1b receptors that are highly expressed in aortic tissues [38]. A potential confounding issue in the use of the Tie2 promoter is that, in addition to effects on endothelial cells, there is also deletion of genes in myeloid cells due to common precursor cells. However, this does not appear to impact the present study based on the previous demonstration that deletion of AT1a receptor in bone marrow-derived cells had no effect on atherosclerosis and AAAs [5], [18].

Endothelial cells are the initial barrier for entry of biochemical or cellular mediators of atherosclerosis *in vivo*. AngII exerts effects on endothelial cells in vitro that may potentially promote atherosclerosis, including increased expression of VCAM-1 [39]; however, this has not been consistently observed [5], [40]. Also, there are several reports of AngII promoting leukocyte adhesion to cultured endothelial cells [5], [40], [41]. Despite strong evidence from in vitro studies that AngII stimulation of endothelial cells is pro-atherogenic, endothelial specific depletion of AT1a receptors had no effects on the development of atherosclerosis in both genders of LDL receptor −/− mice.

The endothelium has a less defined role in the development of AAAs compared to atherosclerosis. We have demonstrated recently that endothelial-specific deletion of AT1a receptors partially attenuates the development of AngII-induced thoracic aortic aneurysms [24]. However, the pathology of AngII-induced aortic aneurysms in the ascending aorta differs markedly from those formed in the abdominal aorta [42]. Indeed it has been proposed that AngII-induced AAAs involve adventitial mechanisms [43], [44]. This proposal would be consistent with AngII not exerting effects on endothelium in AAA formation.

Smooth muscle cells are one of the prominent components of human atherosclerotic lesions [45], [46]. This cell type is a more sparse contributor to the cellularity of lesions in hypercholesterolemic mice, such as LDL receptor −/− and apoE −/− mice [47], [48]. Nevertheless, genetic deletion of some molecules in smooth muscle cells has been shown to influence the development of atherosclerosis in these hypercholesterolemic mouse models. These include deletions of peroxisome proliferator-activated receptor gamma [49], cGMP-dependent protein kinase [50], and low density lipoprotein related protein [51]. Even in the absence of substantial numbers of smooth muscle cells within lesions, there is also possible that AngII stimulation of this cell type in the media could influence lesion formation in the intima. For example, AngII incubation with cultured smooth muscle cells induces expression of MCP-1 [52]. Deficiency of MCP-1 reduces atherosclerotic development, although the *in vivo* cellular sources of this cytokine in promoting lesion formation has not been defined [53]. Our results demonstrate that depletion of AT1a receptors in this cell type had no discernible effect on atherosclerosis in both males and females. Smooth muscle cell-specific deletion of AT1a receptors also had no effect on systolic blood pressure in the presence or absence of AngII infusion, which is consistent with previous reports [24], [54]. The SM22 promoter used to drive Cre synthesis in this study has some vascular regional-specific expression. There is low expression in renal vasculature, the major arterial bed regulating AngII-induced increases in blood pressure [54]. Therefore, the equivalent AngII-induced increases in blood pressure in SM22-Cre expressing and non transgenic littermates are likely due to lack of deletion in pressor vascular beds. In agreement, recent studies demonstrated that AngII-induced elevations in blood pressure are reduced by removal of AT1a receptors from renal proximal tubule cells [55], [56].

AngII-induced AAAs are characterized by local disruption within smooth muscle cells of the media [35]. Therefore, we anticipated a profound role for smooth muscle cell-specific deletion of AT1a receptors in vascular pathology. In a recent study, we were able to detect a partial role of AT1a receptor depletion in smooth muscle cells on AngII-induced AAAs in neonatal female mice that were transiently exposed to testosterone and then infused with the peptide as adults [34]. In the present study, male mice were studied since this gender has a greater propensity to develop AngII-induced AAAs [57], [58]. We were unable to discern any effect of smooth muscle cell-specific depletion of AT1a receptors in large groups of adult male mice for AngII-induced AAAs. Despite lack of defining a direct effect of AngII on smooth muscle cells, there has been evidence of a pronounced role of adventitial fibroblasts in the development of AngII-induced aorta pathologies [44]. Therefore, future experiments examining the cellular basis of AngII-induced AAAs will involve this cell type.

In conclusion, the current study has used recently developed and validated AT1a receptor floxed mice to determine the effects of AT1a receptors in endothelial or smooth muscle cells on the development of atherosclerosis and AAAs in LDL receptor −/− mice. Although whole body deletion of this receptor in these mice caused pronounced decreases in atherosclerosis and AAAs [15], [18], deletion of AT1a receptors in either endothelial or smooth muscle cells had no discernible effect on atherosclerotic lesion size or AAA formation.

## Supporting Information

Figure S1 **Genotyping of experimental mice for AT1a receptor floxed allele and Cre transgene by PCR.** Genomic DNA from tail biopsies was isolated and screened by PCR for: (**A**) wild type and floxed AT1a receptor alleles and, (**B**) Cre transgene using IL-2 gene are control. Reaction products were sized using agarose gel electrophoresis. The primer sets and predicted product size are listed below.
(TIF)

Figure S2 **Depletion of AT1a receptors in endothelial cells had no effects on lipoprotein distribution of cholesterol.** Serum (50 µl) from individual mice (n = 3−4/group; A =  males; B =  females) was resolved by size exclusion chromatography. Symbols are group means and bars are standard error of the means.
(TIF)

Figure S3 **Depletion of AT1a receptors in smooth muscle cells had no effects on lipoprotein distribution of cholesterol.** Serum (50 µl) from individual mice (n = 3−4/group; A =  males; B =  females) was resolved by size exclusion chromatography. Symbols are group means and bars are standard error of the means.
(TIF)
