# Supplementary figures and images for: Depletion of Endothelial or Smooth Muscle Cell-Specific Angiotensin II Type 1a Receptors Does Not Influence Aortic Aneurysms or Atherosclerosis in LDL Receptor Deficient Mice
Source: PLoS One. 2012 Dec 7;7(12):e51483. doi: 10.1371/journal.pone.0051483 (PMC3517567; doi:10.1371/journal.pone.0051483)

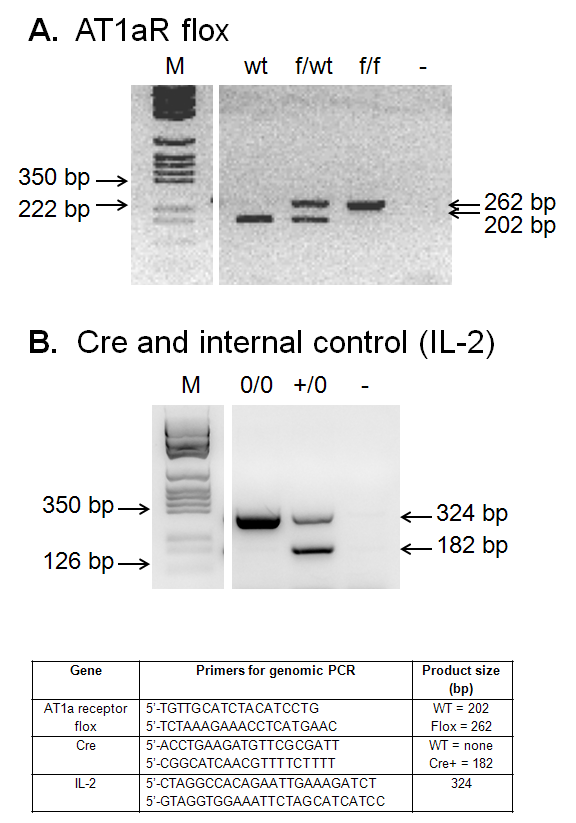

Supplement: Figure S1 — Genotyping of experimental mice for AT1a receptor floxed allele and Cre transgene by PCR. Genomic DNA from tail biopsies was isolated and screened by PCR for: (A) wild type and floxed AT1a receptor alleles and, (B) Cre transgene using IL-2 gene are control. Reaction products were sized using agarose gel electrophoresis. The primer sets and predicted product size are listed below. (TIF) [file pone.0051483.s001.tif]

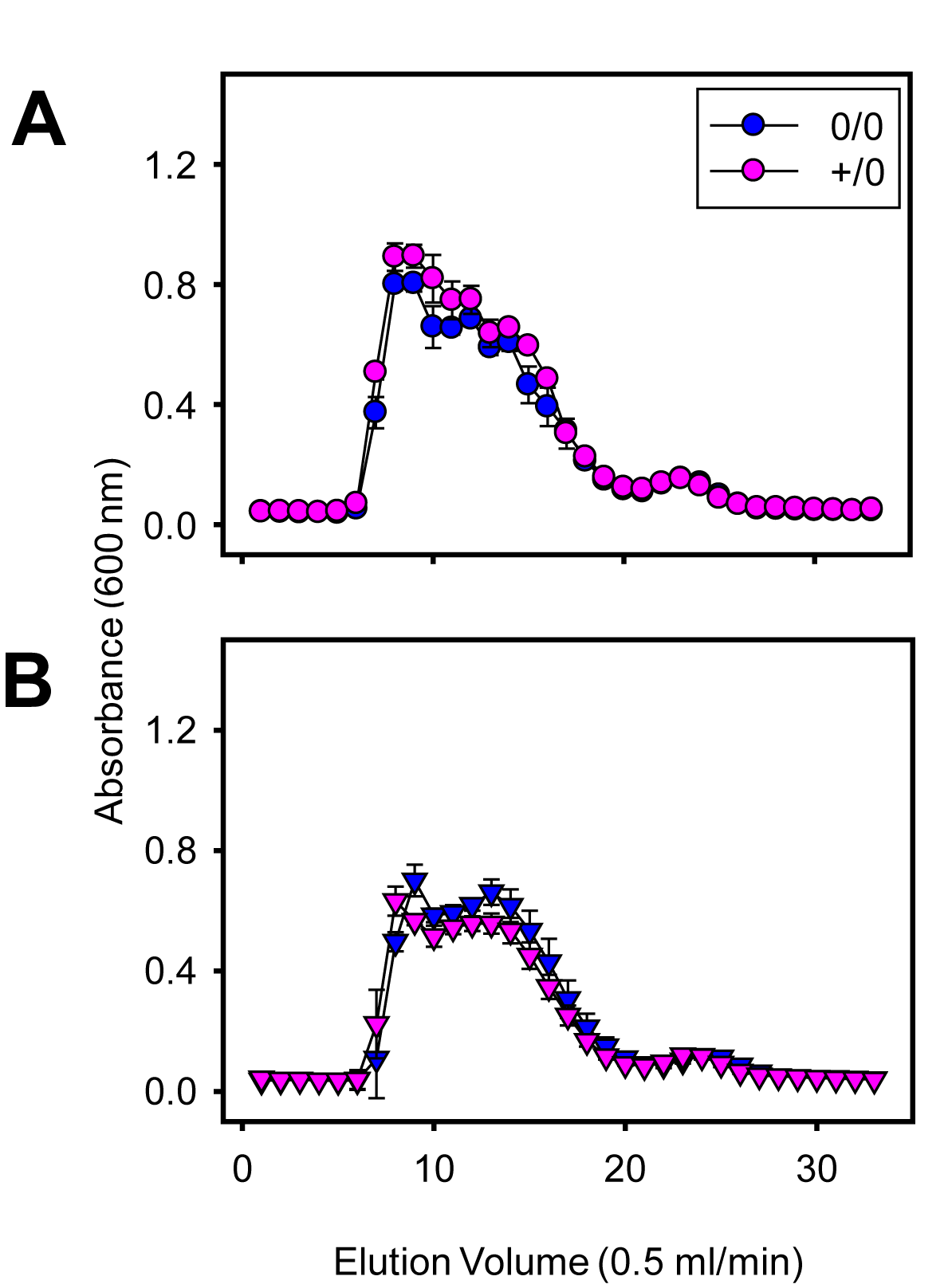

Supplement: Figure S2 — Depletion of AT1a receptors in endothelial cells had no effects on lipoprotein distribution of cholesterol. Serum (50 µl) from individual mice (n = 3−4/group; A = males; B = females) was resolved by size exclusion chromatography. Symbols are group means and bars are standard error of the means. (TIF) [file pone.0051483.s002.tif]

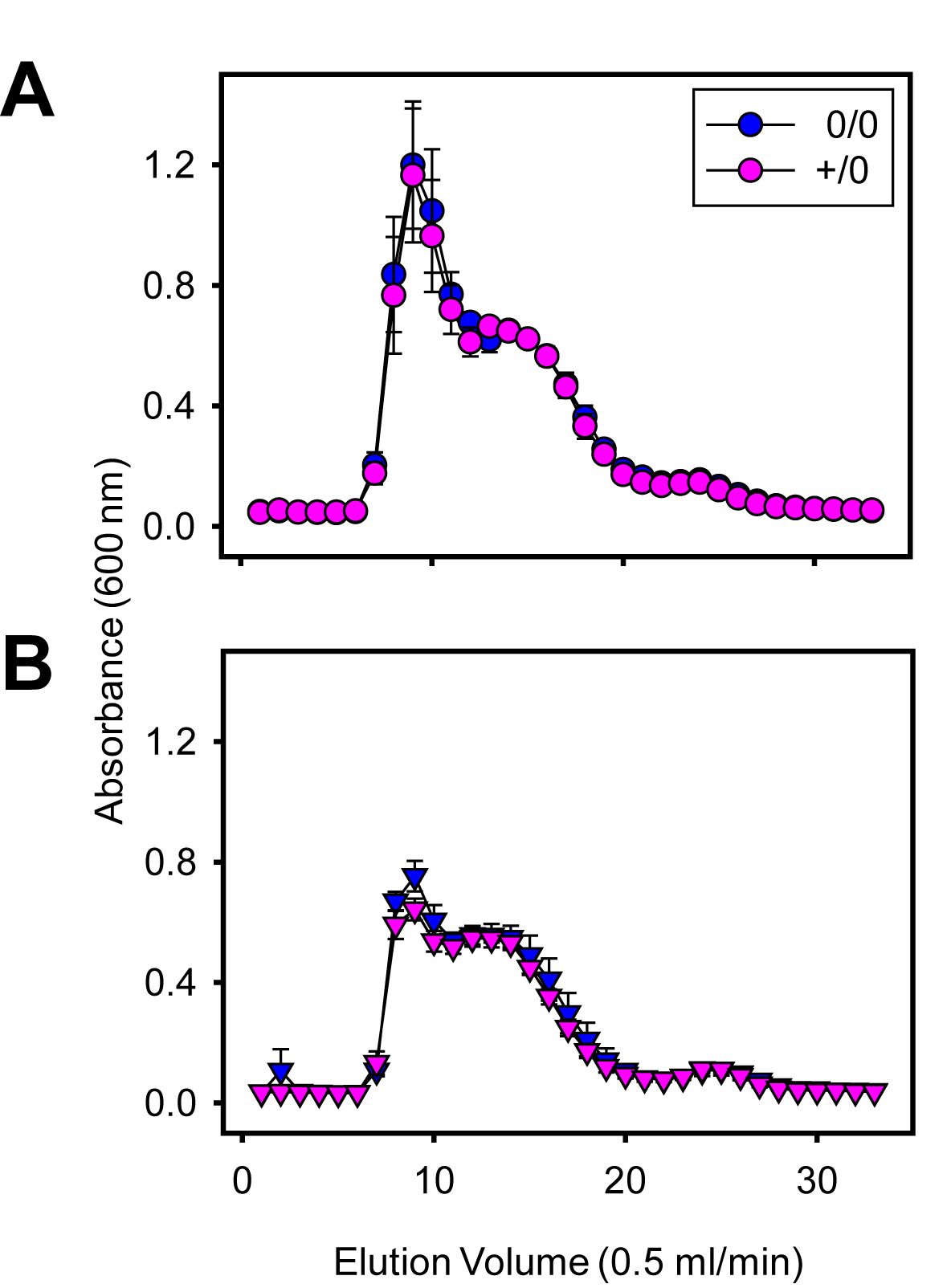

Supplement: Figure S3 — Depletion of AT1a receptors in smooth muscle cells had no effects on lipoprotein distribution of cholesterol. Serum (50 µl) from individual mice (n = 3−4/group; A = males; B = females) was resolved by size exclusion chromatography. Symbols are group means and bars are standard error of the means. (TIF) [file pone.0051483.s003.tif]
